# Medicine of the Nineteenth Century

**Published:** 1901-03

**Authors:** 


### Abstracts and Translations.

### MEDICINE OF THE NINETEENTH CENTURY.

NOTWITHSTANDING the power that the discovery of the uses of steam and electricity has given to the race; notwithstanding the happiness that education and improved ethics have brought to humanity, the greatest gift that the nineteenth century has given to man has been the discovery of scientific laws that lead to health; laws by the knowledge of which the plague and the diseases of other countries have been kept from spreading through the community, and laws which have taught physicians how to speedily ease suffering and to save and lengthen life.

A masterly and comprehensive syndicate article on "Medicine in the Nineteenth Century," from the pen of Dr. Osler, has just appeared. In it he sums up the discoveries and the advances of medical science in the last one hundred years, and to the layman his lucid descriptions of progress cannot but be a revelation.

At the beginning of the last century the theory that "aqueous" and "dry humors" were the seat of disease was still taught. In 1801 Bichat, a young French physician, taught that the seat of disease was not in the organs themselves, but in the tissues of the organs, and thus gave an impulse to the study of morbid anatomy. Laennec discovered the art of auscultation, and his contributions to the study of the diseases of the lung and heart really laid the foundation of modern clinical medicine. Bright's researches on the kidneys and the differentiation of the fevers into typhoid, typhus, relapsing, and yellow were the beginning of the original work. Once the idea of scientific investigation took the place of the philosophic discussion of theories, the light began to break, and as it lit up one source of disease after another, we, who look backward in review, can see how dense must have been the darkness of ignorance before the present century.

In the growth of specialism, while there has, perhaps, been a loss in breadth and harmony, there has been the compensation of greater accuracy in the application of special knowledge. This has been particularly valuable in the diseases which are peculiar to women, especially to accidents incident to child-bearing, and in the great improvement in the treatment of insanity.

At the beginning of the century there were but three medical colleges in the United States and only two general hospitals. It was the custom of practitioners to take students as apprentices, and the well-to-do finished their education abroad. After 1830 a remarkable change took place, owing to the leaven of French science brought from Paris by American students. Between 1840 and 1870 there was a great increase in the number of medical schools, but the standard was low and the work poor, from the multiplicity and rivalry of ill-equipped institutions. However, the evolution of the medical school that followed the reformation, begun in 1868, by raising the standard over the whole country, was one of the most remarkable phenomena in the history of medicine in the United States.

Dr. Osler calls attention to the fact that sanitary science, or hygiene, is one of the bright spots of the century. A knowledge of the conditions of disease, and the means of transmission together with the culmination of the art of preventive measures, has made small-pox, as a dangerous disease, a thing of the past. It has effectually kept cholera and the bubonic plague from spreading in the country. It has restricted yellow fever to its original localities, and has reduced the death-rate of typhus from twelve hundred and twenty-eight per million—in England sixty years ago—to three per million to-day, while it has so conclusively proved the exact precautions that must be observed to prevent typhoid and tuberculosis, that it is only through gross carelessness and wilful lack of sanitary measures that they can spread.

But as he remarks: "Preventive medicine was a blundering, incomplete science until bacteriology opened unheard-of possibilities for the prevention of disease." The brilliant overthrow of the hitherto much-discussed theory of "spontaneous generation," by Pasteur, Tyndall, Koch, and Cohn, and the tracing of each disease to its bacteriologic origin has been the bright triumph of the century. It has led to a knowledge of wound infection, which has served as the foundation stone of the science and art of modern surgery. It has been the means of determining definitely the cause of leprosy, so that plague may never again obtain a foothold. It has reduced the aggregate death-rate of diphtheria fully fifty per cent. since the discovery of antitoxin, and concerning that most deadly and insidious of human diseases, which Dr. Oliver Wendell Holmes called the "white plague,"—tuberculosis,—it has taught the origin and means of prevention, that may, if faithfully followed, lead to the eradication of the disease. In summing up the revelations that have been made concerning contagious disease, through bacteriologic investigation, Dr. Osler outlines the fields of investigation that have not yet been worked with success. The cause of contagion of the exanthemata, measles, scarlet fever, chicken-pox, small-pox, and of whooping-cough, though yet unknown, he thinks may be of bacterial origin, but that the special bacteria may be too minute for any known microscope to reveal.

The last word of the century on these lines is concerning serum-therapy, which experimenters are trying for every contagious disease, with effects that will probably modify present lines of treatment within the next ten years. While there has been a remarkable diminution in the prevalence of a large number of all the acute infections, "pneumonia," says Dr. Osler, "not only holds its own, but seems to have increased in its virulence," while concerning venereal diseases, which continue to embarrass the social economist and to perplex and distress the profession, he recommends legislation.

The brilliant experiments of the summer of 1900 on the Roman Campagna, proving conclusively that malaria can be communicated by mosquitoes, and solving at once the mystery of marshy lands, the hot season, and the night air, is almost a spectacular ending to the century's work and opens an entirely new field for investigation concerning the relations of insects and animals to human diseases.

One of the striking features of modern medicine, as Dr. Osler points out, is the tendency on the part of physicians to give little or no medicine and to substitute attention to diet, exercise, rest, and climate for drugs. And yet the century never witnessed a more perfect and all-abiding faith in drugs on the part of the layman than at its close. But as this man of wisdom concludes, faith is as ever a large element in the success of the practitioner, and, as Galen said, "confidence and hope do more than physic."—*Editorial in The Journal of the American Medical Association.*

